# Identification of Carcinogenesis and Tumor Progression Processes in Pancreatic Ductal Adenocarcinoma Using High-Throughput Proteomics

**DOI:** 10.3390/cancers14102414

**Published:** 2022-05-13

**Authors:** Lucía Trilla-Fuertes, Angelo Gámez-Pozo, María Isabel Lumbreras-Herrera, Rocío López-Vacas, Victoria Heredia-Soto, Ismael Ghanem, Elena López-Camacho, Andrea Zapater-Moros, María Miguel, Eva M. Peña-Burgos, Elena Palacios, Marta de Uribe, Laura Guerra, Antje Dittmann, Marta Mendiola, Juan Ángel Fresno Vara, Jaime Feliu

**Affiliations:** 1 Molecular Oncology & Pathology Laboratory, Instituto de Genética Médica y Molecular-INGEMM, Hospital Universitario La Paz-IdiPAZ, 28046 Madrid, Spain; lucia.trilla@salud.madrid.org (L.T.-F.); angelo.gamez@salud.madrid.org (A.G.-P.); mariaisabell.lh@gmail.com (M.I.L.-H.); rlvacas@gmail.com (R.L.-V.); juanangel.fresno@salud.madrid.org (J.Á.F.V.); 2 Molecular Pathology and Therapeutic Targets Group, Hospital Universitario La Paz-IdiPAZ, 28046 Madrid, Spain; victoriam.heredia@salud.madrid.org (V.H.-S.); maria.miguel@salud.madrid.org (M.M.); marta.mendiola@salud.madrid.org (M.M.); 3 Biomedical Research Networking Center on Oncology-CIBERONC, ISCIII, 28029 Madrid, Spain; 4 Medical Oncology Service, Hospital Universitario La Paz, 28046 Madrid, Spain; isma_g_c@hotmail.com; 5 Biomedica Molecular Medicine SL, 28049 Madrid, Spain; elena.lopez@biomedicamm.com (E.L.-C.); andrea.zapater@biomedicamm.com (A.Z.-M.); 6 Pathology Department, Hospital Universitario La Paz, 28046 Madrid, Spain; evamanuel.pena@salud.madrid.org (E.M.P.-B.); mariaelena.palacios@salud.madird.org (E.P.); marta.deuribe@salud.madrid.org (M.d.U.); laura.guerra@salud.madrid.org (L.G.); 7 Functional Genomics Center Zurich, University of Zurich/ETH Zurich, 8057 Zurich, Switzerland; antje.dittmann@fgcz.ethz.ch; 8 Cátedra UAM-ANGEM, Faculty of Medicine, Universidad Autónoma de Madrid, 28046 Madrid, Spain

**Keywords:** pancreatic ductal adenocarcinoma, high-throughput proteomics, carcinogenesis, tumor progression, molecular profiles

## Abstract

**Simple Summary:**

Pancreatic cancer is an aggressive disease with a high mortality rate. The study of the biological processes involved in carcinogenesis (tumor formation) and tumor progression (development of metastases) is still necessary. In this work, we established three subtypes of pancreatic tumors according to their protein profiles: one adhesion subtype, a metabolic subtype, and a nucleoplasm subtype. In addition, the identified mechanisms involved in carcinogenesis and in tumor progression differ between subtypes. These differences may need to be considered when designing new treatments.

**Abstract:**

Pancreatic ductal adenocarcinoma (PDAC) is an aggressive disease with an overall 5-year survival rate of just 5%. A better understanding of the carcinogenesis processes and the mechanisms of the progression of PDAC is mandatory. Fifty-two PDAC patients treated with surgery and adjuvant therapy, with available primary tumors, normal tissue, preneoplastic lesions (PanIN), and/or lymph node metastases, were selected for the study. Proteins were extracted from small punches and analyzed by LC-MS/MS using data-independent acquisition. Proteomics data were analyzed using probabilistic graphical models, allowing functional characterization. Comparisons between groups were made using linear mixed models. Three proteomic tumor subtypes were defined. T1 (32% of patients) was related to adhesion, T2 (34%) had metabolic features, and T3 (34%) presented high splicing and nucleoplasm activity. These proteomics subtypes were validated in the PDAC TCGA cohort. Relevant biological processes related to carcinogenesis and tumor progression were studied in each subtype. Carcinogenesis in the T1 subtype seems to be related to an increase of adhesion and complement activation node activity, whereas tumor progression seems to be related to nucleoplasm and translation nodes. Regarding the T2 subtype, it seems that metabolism and, especially, mitochondria act as the motor of cancer development. T3 analyses point out that nucleoplasm, mitochondria and metabolism, and extracellular matrix nodes could be involved in T3 tumor carcinogenesis. The identified processes were different among proteomics subtypes, suggesting that the molecular motor of the disease is different in each subtype. These differences can have implications for the development of future tailored therapeutic approaches for each PDAC proteomics subtype.

## 1. Introduction

Pancreatic ductal adenocarcinoma (PDAC) is an aggressive disease with an overall 5-year survival rate of only 5%. At the time of diagnosis, 80% of tumors are already in incurable stages. On the other hand, in patients with localized disease, surgery represents the best option of curative treatment as part of multidisciplinary management, including chemotherapy and radiation therapy. However, despite performing a radical resection, 80% of patients are going to relapse [1,2]. In 2021, 60,430 new PDAC cases and 48,220 related deaths have been estimated in the United States with an increasing incidence, being the fourth cause of cancer death [3]. Therefore, it is an absolute priority to deepen the knowledge of PDAC pathogenesis.

PDAC molecular subtypes have already been defined using transcriptomics data [4,5,6]. Collison et al. divided PDAC into a classical, exocrine-like, and quasi-mesenchymal subtype [5]. Moffit et al. established a classification making distinctions between tumor subtypes -basal-like and classical-, and stromal subtypes—normal and activated [4]. Finally, Bailey et al. divided PDAC into squamous, pancreatic progenitor, immunogenic, and aberrantly differentiated endocrine exocrine (ADEX) subtypes [6]. Squamous, quasi-mesenchymal, and basal-like subtypes are well aligned across the three classifications. Puleo et al. suggested that the differences shown by Bailey et al. were due to the cellularity of the samples [7].

Proteomics has been developed as a complementary approach to the massive sequence of genes and genomes and to analysis at the RNA level. Its importance lies in the fact that proteins ultimately define the function and the operations of cells, tissues, and organisms [8]. Whereas genomics usually shows why things happen, proteomics explains what is happening. In this regard, genomics and proteomics complement each other, integrating different levels of information.

A previous study defined proteomics subtypes of PDAC using hepatic metastases, classifying tumors into metabolic, progenitor-like, proliferative, and inflammatory subtypes [9]. Another study identified four risk subgroups of PDAC using proteomics [10]. Recently, Cao et al. studied early biomarkers of PDAC by proteogenomics using tumors and normal adjacent tissues [11]. However, until now, a proteomics study to define those processes involved in tumor development and progression has not been performed.

In this study, a molecular characterization of paired PDAC samples (normal tissue-preneoplastic lesions-primary tumor-lymph node metastases) based on a proteomics analysis pipeline followed by computational approaches was performed to deepen the molecular information.

Coupling proteomics with our data analysis pipeline allows the identification of those biological processes related to carcinogenesis and tumor progression through the analysis of paired samples. Network analysis based on probabilistic graphical models (PGMs) was used to further characterize those biological functions that may be relevant to tumor development and progression by comparing the different types of samples. Three proteomics tumor PDAC subtypes were identified, and biological processes involved in carcinogenesis and tumor progression were different among them.

## 2. Materials & Methods

### 2.1. Patient Samples and Clinical Data

Patients with PDAC treated with surgery and adjuvant therapy from February 2010 to October 2020 at Hospital Universitario La Paz with available FFPE primary tumor and normal tissue, preneoplastic lesions grade 2–3 (PanIN), and/or lymph node metastases were selected for the study. Samples were punched to study the differences associated with the different types of regions. A total of 52 primary tumors, 47 non-tumor tissues, 43 PanIN, and 31 lymph nodes were obtained for the proteomics analyses. The study was approved by the Ethical Committee of the Hospital Universitario La Paz (IRB number: 1349). The 7th edition of TNM was used to classify the samples.

### 2.2. Protein Isolation

Protein isolation was performed as previously described [12]. Briefly, the FFPE sections were deparaffinized in xylene and washed twice in absolute ethanol. Protein isolates were prepared in 2% SDS. Protein quantity was measured using a MicroBCA Protein Assay Kit (Pierce-Thermo Scientific). Finally, 10 µg of each protein extract were digested with trypsin (1:50), and SDS was eliminated from the lysates using Detergent Removal Spin Columns (Pierce). Before mass-spectrometry experiments, samples were desalted using ZipTips (Millipore), dried, and resolubilized in 15 µL of a 0.1% formic acid and 3% acetonitrile solution.

Peptides were acidified to perform a stage-tip cleanup using two Empore reversed-phase extraction disks (3M) [13]. Digests were dried in a SpeedVac and stored at −20 °C until LC-MS/MS analysis. Peptides were re-solubilized in 20 µL of 3% acetonitrile, 0.1% formic acid, and 1 µL of indexed retention time (iRT)-peptides (Biognosys) were spiked in each sample for MS analysis. For the DDA analysis and subsequent spectral library generation, a small volume of each sample was taken and combined into a total of 10 pooled samples.

### 2.3. Liquid Chromatography–Mass Spectrometry Experiments

One hundred and seventy-three samples from 52 PDAC patients, including non-tumor tissue, primary tumor, PanIN, and affected lymph nodes, were analyzed by high-throughput proteomics.

Mass spectrometry analysis was performed on an Orbitrap Fusion (Thermo Scientific) equipped with a Digital PicoView source (New Objective) and coupled to an M-Class UPLC (Waters). The solvent composition of the two channels was 0.1% formic acid for channel A and 0.1% formic acid, 99.9% acetonitrile for channel B. For each sample, 2 μL of peptides were loaded on a commercial MZ Symmetry C18 Trap Column (100 Å, 5 µm, 180 µm × 20 mm, Waters) followed by a nanoEase MZ C18 HSS T3 Column (100 Å, 1.8 µm, 75 µm × 250 mm, Waters). The peptides were eluted at a flow rate of 300 nL/min. After an initial hold at 5% B for 3 min, a gradient from 5% to 22% B in 109 min and 32% B in 8 min was applied. The column was washed with 95% B for 5 min, and afterwards the column was re-equilibrated to starting conditions for an additional 10 min.

For library generation using the pooled samples, the mass spectrometer was operated in data-dependent mode (DDA), acquiring a full-scan MS spectra (350−1500 *m*/*z*) at a resolution of 120,000 at 200 *m*/*z* after accumulation to a target value of 400,000. Data-dependent MS/MS were recorded in the Orbitrap using quadrupole isolation with a window of 1.4 Da and HCD fragmentation with 30% normalized collision energy (NCE). Orbitrap resolution was set to 30,000, maximum injection time was set to 54 ms with a target value of 50,000, and the cycle time was set to 3 s. Charge-state screening was enabled. Singly, unassigned, and charge states higher than seven were rejected. Precursor masses previously selected for MS/MS measurement were excluded from further selection for 25 s, and the exclusion window was set at 10 ppm.

For the analysis of the individual samples, the mass spectrometer was operated in a data-independent mode (DIA). The DIA scans covered a range from 400 to 1100 *m*/*z* in windows of 20 *m*/*z*. The resolution of the DIA windows was set to 30,000, with an AGC target value of 50,000, the maximum injection time set to Dynamic, and a NCE of 30. Each instrument cycle was completed by full MS scan monitoring 350 to 2000 *m*/*z* at a resolution of 120,000.

The samples were acquired using internal lock mass calibration on m/z 371.1010 and 445.1200. The mass spectrometry proteomics data were handled using the local laboratory information management system (LIMS) [14], and all relevant data were deposited to the ProteomeXchange Consortium via the PRIDE (http://www.ebi.ac.uk/pride) partner repository with the data set identifier PXD032076.

### 2.4. Spectral Library Generation and Protein Quantification

A hybrid spectral library was generated using the Pulsar search engine and spectral library generation functionality in Spectronaut (14.0.200601.47784, Biognosys), applying the default parameter settings to DDA and DIA runs. Spectra were searched against a canonical SwissProt database for human and common protein contaminants (NCBI taxonomy ID9606, release date 9 July 2019). Carbamidomethylation of cysteine was set as a fixed modification, while methionine oxidation and N-terminal protein acetylation were set as variable modifications. Enzyme specificity was set to trypsin/P allowing a minimal peptide length of 7 amino acids and a maximum of two missed-cleavages. Precursor and fragment tolerance were set to Dynamic for the initial search. The maximum false discovery rate (FDR) was set to 0.01 for peptides and 0.01 for proteins. Protein quantification was performed in Spectronaut using default settings. The quantitative data were extracted using the BGS Factory Report (default) and used for follow-up analyses. Stringent filtering of the extracted feature groups by the Spectronaut-reported q-Value was applied. For precursor fragment groups, we required a per run q-value of at most 0.05 and a per experiment q-value of at most 0.01. The q-value sparse mode was used in combination with a global imputing strategy. To perform statistical modeling, fragment intensities were aggregated into precursor and peptide intensities.

### 2.5. Data Preprocessing

Proteomics data were transformed into log2. At least 75% of valid values in at least one group (non-tumor tissue, PanIN, primary tumor, and lymph nodes) were applied as quality criteria. Then, missing values were imputed to a normal distribution using Perseus software [15].

### 2.6. Study of GATA6 Expression by Immunohistochemistry

For GATA6 determination, optimal tissue blocks were selected by an expert pathologist on hematoxylin and eosin (H&E) slides. Representative tumor areas of each case were selected for tissue microarray (TMA) construction. Two representative cores of 1.2 mm in diameter were taken and arrayed into a receptor block using a tissue microarray (TMA) workstation (Beecher Instruments, Silver Spring, MD, USA) as previously described [16]. Four micrometer sections of the TMAs were used for immunohistochemistry (IHC) purposes. Briefly, slides were cut with a semiautomatic microtome HM 3508 (MICROM), deparaffinized, and rehydrated in water. Antigen retrieval was performed using a DAKO PT Link. Peroxidase activity was blocked with a Dako Protein block for 10 min, incubated for 30 min with primary antibodies, detected with a Dako Envision Plus kit, and counterstained with haematoxylin. All reagents were from Dako (Agilent, Santa Clara, CA, USA). GATA-6 antibody used: ref. number AF1700 (R&D Systems, MN, USA).

### 2.7. Probabilistic Graphical Models

As in previous works [12,17], probabilistic graphical models (PGMs) were calculated using proteomics data without any a priori information in R using the *grapHD* package [18]. This analysis allows the organization of protein data according to their expression profile and the identification of relevant biological processes. The resulting networks were split into functional nodes according to the gene ontology of their branches. Gene ontology analyses to assign a function to each functional node were done in DAVID webtool [19], using homo sapiens as background and GOTERM-FAT, Biocarta, and KEGG as categories. Once the functional nodes were assigned, functional node activities were calculated as the mean expression of those proteins involved in the main function of each node. These functional node activities were used to make comparisons between groups of samples.

### 2.8. Statistical Analyses

Hierarchical cluster (HCL) based on correlation and average linkage to establish tumor proteomics subtypes was done using MeV software [20]. Mixed linear models with fixed effects were used to establish the significant differences between the groups of samples. These calculations were done in R using library lme4 [21]. For the comparison between tumor samples, a Mann–Whitney test was used. Finally, the relationships between clinical parameters and subtypes were studied using Chi-squared tests. These tests were done using Graph Pad Prism v6; *p*-values were two-sided and considered significant below 0.05.

### 2.9. Validation of PDAC Proteomics Subtypes in the TCGA Cohort

A centroid based on the 313 differential proteins defined in the SAM was calculated for each tumor proteomic subtype. On 284 of these 313 proteins, an equivalent gene existed in the TCGA cohort. Using these 284 genes, TCGA samples were classified into one of the three defined subtypes. Then, functional node activities were calculated to verify that the subgroups had the same molecular features as the tumor proteomics subtypes.

## 3. Results

### 3.1. Clinical Data

From a cohort of 110 PDAC patients treated with surgery and adjuvant therapy from February 2010 to October 2020 at Hospital Universitario La Paz, fifty-two patients were selected for proteomics experiments. For these patients, all the available samples were analyzed: non-tumor tissue, preneoplastic lesions grade 2–3 (PanIN), tumor, and lymph nodes. Fourty-seven non-tumor samples, 43 PanIN, and 31 lymph node samples were available.

Regarding clinical data, only information of 50 patients was available due to loss of follow-up after surgery of two of them. These 50 patients were used for the analyses that involved clinical parameters (Table 1).

The median follow-up was 13 months, and 37 relapses occurred, of which 10 were local relapses and 27 were distant relapses. All patients were treated with surgery and adjuvant therapy, and none received neoadjuvant therapy.

### 3.2. Proteomics Experiments

A total of 3927 proteins were identified in DIA mass-spectrometry experiments. After applying a quality criterion of at least 75% of valid values in at least one group (non-tumor tissue, PanIN, primary tumor, and lymph nodes), 2311 proteins were used for the subsequent analyses.

### 3.3. Proteomics Pancreatic Dutal Adenocarcinoma Subtypes

First, all the samples were analyzed by a hierarchical cluster (HCL) to establish differences between different types of tissues. Surprisingly, the HCL was not capable of splitting samples by tissue type, i.e., establishing a group of non-tumor tissue, another of tumor samples, another with PanIN and a last one containing the lymph node samples (Figure S1).

**Figure 1:**
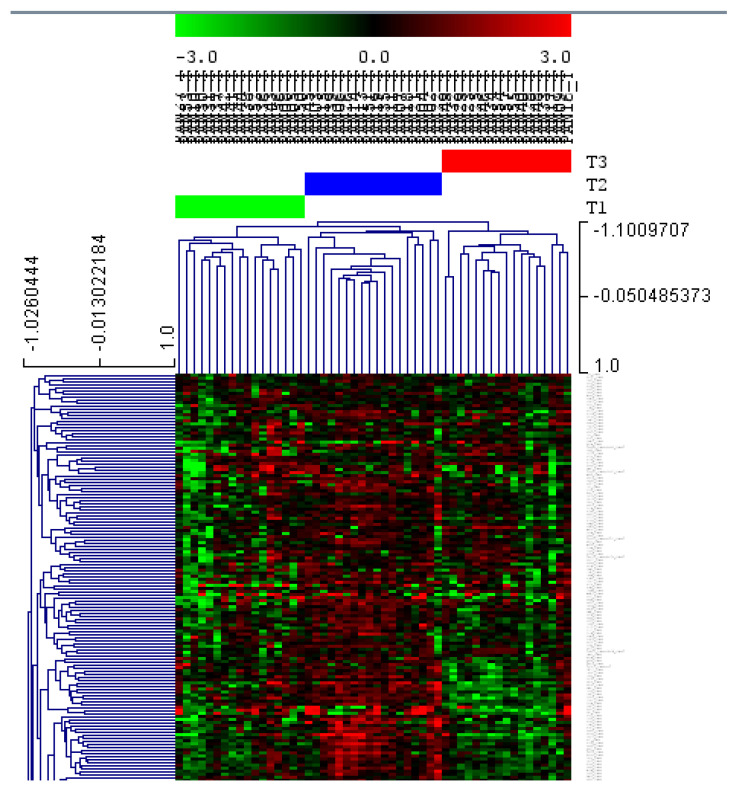
Hierarchical clustering (HCL) of PDAC tumor samples clearly showed three proteomic subtypes (T1, T2, and T3). HCL is based on the average linkage method and Pearson correlation.

In order to establish whether the variability associated with this distribution that does not distinguish by sample origin was related to different proteomics tumor subtypes, only tumor samples were selected to perform the analysis. In this case, the HCL clearly established three different groups of tumors in PDAC according to their proteomic profiles. T1 included 16 (32%) patients, whereas T2 and T3 were composed of 17 (34%) patients each (Figure 1).

**Figure 2:**
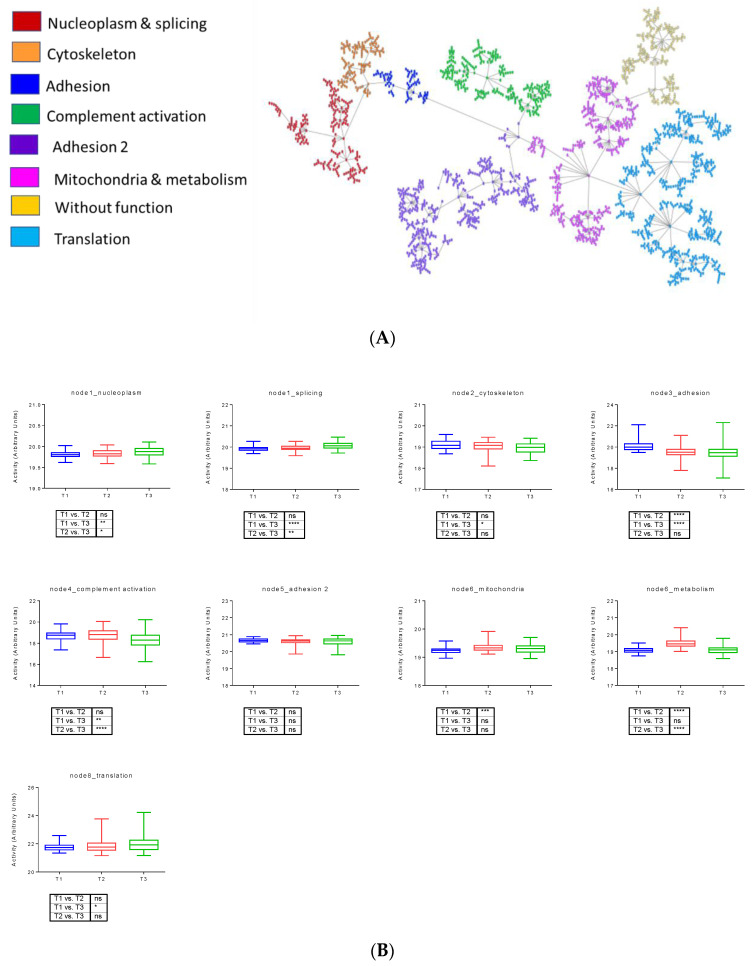
(**A**) Network formed by 2311 proteins in PDAC tumor samples. (**B**) Functional node activities comparing the three proteomic subtypes in tumor samples. **** *p* < 0.0001; *** 0.0001 < *p* < 0.001; ** 0.001 < *p* < 0.05. * *p* < 0.05; ns: nucleoplasm subtype.

As in previous works [12,17], a network analysis based on PGMs was used to characterize in depth the differences at the biological processes level between the three proteomics PDAC tumor subtypes. The resulting network was divided into eight functional nodes, two of which had an overrepresentation of adhesion proteins. Functional node activities showed differences between the three subtypes. T1 presented higher functional node activities in adhesion and complement activation nodes, and will be referred as “adhesion subtype” for now on. T2 had higher functional node activities of mitochondria and metabolism and translation nodes, being for now on the “metabolic subtype”. Finally, T3 showed higher functional node activities in nucleoplasm and splicing, and will be named as “nucleoplasm subtype” (Figure 2).

Regarding the clinical relevance of these subtypes, T1 and T2 contained most of the pancreatic tumors located in the head of the pancreas, and T3 contained most of the tumors located in the body and tail (Supplementary Figure S1A). There were no significant differences between T groups according to age, gender, diabetes, pancreatitis, smoking, grade, type of resection, pT, pN, stage, or location of metastases. Any differences in prognosis according to overall survival or disease-free survival between the three PDAC proteomics subtypes were found (Supplementary Figure S1B). The percentage of relapses at 12 months was 37% in the T1 subtype and 53% in the T2 and T3 subtypes.

### 3.4. Study of Classical Defined Biomarkers from PDAC Transcriptomics Subtypes

Of the defined biomarkers from transcriptomics PDAC subtypes, only Mucin 5 (MUC5A), characteristic of Moffit classical subtype and Bailey progenitor subtype, and insulin (INS), characteristic of Bailey’s ADEX subtype, were identified in the list of the identified and quantified proteins. MUC5A expression was compared across the defined PDAC proteomics subtypes, and it was significantly higher in the T1-adhesion subtype. Additionally, insulin protein (INS) had a higher expression in the T2 subtype, being comparable with the ADEX subtype (Supplementary Figure S2).

GATA6, a marker characteristically expressed in the Moffit classical subtype, was studied by IHQ. All T3 tumors showed a positive expression of GATA6 by IHC, and negative ones were split into T1 and T2 subtypes (Supplementary Figure S3). Altogether, these results suggested that T2 tumors correspond to the ADEX subtype and contained classical and basal-like tumors; T1 also contained basal-like and classical subtypes; and T3 corresponded only to classical tumors that also had an overexpression of proteins related to nucleoplasm.

### 3.5. Identification of Biological Processes Involved in Carcinogenesis and Tumor Progression in Each PDAC Proteomics Subtype

Since differences between subtypes are bigger than differences between the types of samples, we studied the differences between samples at a functional level in order to characterize the biological processes involved in the progression of the disease independently in each defined proteomics subtype. Thus, new analyses including each type of sample (normal pancreatic tissue, PanIN, tumor, and lymph nodes) were performed for each proteomics subtype.

#### 3.5.1. Identification of T1 Carcinogenesis and Tumor Progression Processes

T1 tumor samples are characterized by higher adhesion and complement functional node activities compared with the other PDAC proteomics tumor subtypes.

**Figure 3:**
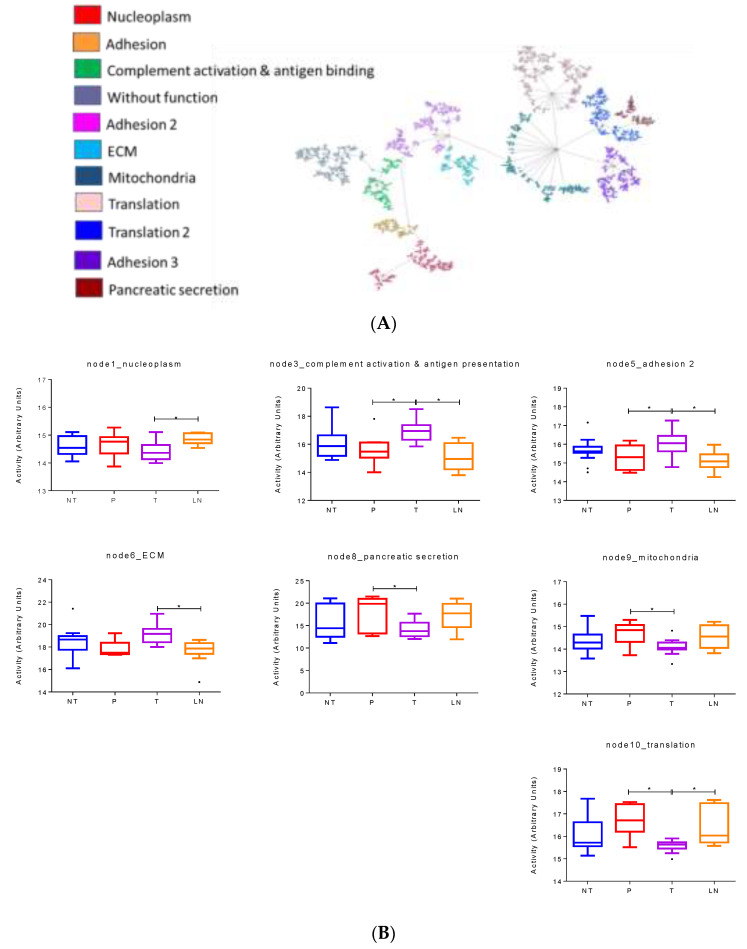
(**A**) Network of 2311 proteins in the T1 subtype. (**B**). Differential functional node activities comparing the different histological samples in the T1 subtype according to mixed linear models. NT = normal tissue, P = preneoplastic lesions, T = primary tumors, LN = lymph nodes. * *p* < 0.05.

A network based on PGMs was constructed, including all types of samples from patients with T1 tumors. The resulting network had 11 functional nodes, one without an overrepresented biological function (Figure 3A). Functional node activities and mixed linear models were used to define the biological processes with differential functional node activities between tissue samples (Figure 3B, Table 2).

Using mixed linear models, differences between non-tumor and tumor tissue were identified. Mitochondria, pancreatic secretion, and translation node activity decreased in tumor samples compared to PanIN, and adhesion2, and complement activation and antigen presentation node activity presented an increase in tumor samples compared to PanIn.

Significant differences between tumors and lymph nodes and, therefore, related to tumor progression were identified in complement activation and antigen presentation, adhesion 2, ECM, nucleoplasm, and translation functional nodes. In this case, nucleoplasm and translation were higher in lymph nodes, and the others suffered a decrease in their activity in lymph node samples.

The adhesion 2 node contains some relevant proteins, such as HSPB1 or THY. Complement activation node was mainly formed by immunoglobulins and complement proteins such as C3 or C1QB. The translation node was mainly formed by ribosomal proteins (RPL3, RL23A, RL11, RL8, RS9, etc.). Finally, the most relevant protein included in the nucleoplasm node was HIF1AN.

#### 3.5.2. Identification of T2 Carcinogenesis and Tumor Progression Processes

**Figure 4:**
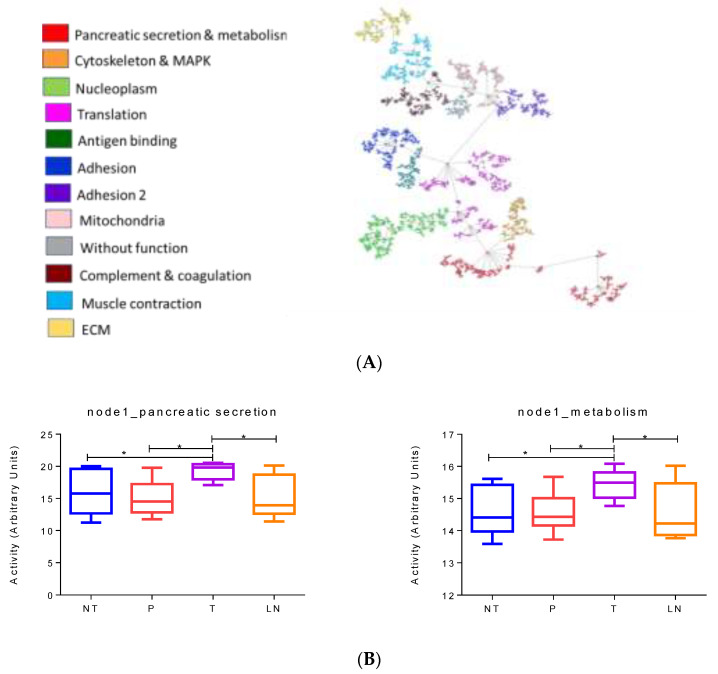
(**A**). Network of 2311 proteins in the T2 subtype. (**B**) Differential functional node activities comparing the different histological samples in the T2 subtype according to mixed lineal models. NT = normal tissue, P = preneoplastic lesions, T = primary tumors, LN = lymph nodes. * *p* < 0.05.

T2 tumors were characterized as having higher mitochondria, metabolism, and translation activity compared to the other PDAC tumor subtypes and presented overlapping characteristics with the ADEX subtype. Again, a network was built using all T2 samples. It was composed of 12 functional nodes, two of which had two associated functions: pancreatic secretion and metabolism, and the other cytoskeleton and MAPK (Figure 4A).

Functional node activities and mixed linear models were used to define those biological processes with differential functional node activity between tissue samples (Figure 4B).

The biological processes identified as related to tumor development were pancreatic secretion and metabolism, which were significantly higher in tumor samples than in normal tissues and PanIN.

These functional node activities (pancreatic secretion and metabolism) presented a significant decrease in lymph node samples compared to tumors, which could be associated with tumor progression.

Pancreatic secretion node contained several relevant proteins such as TYMP, NAMPT, or pancreatic lipases, such as PNLIP.

#### 3.5.3. Identification of T3 Carcinogenesis and Tumor Progression Processes

**Figure 5:**
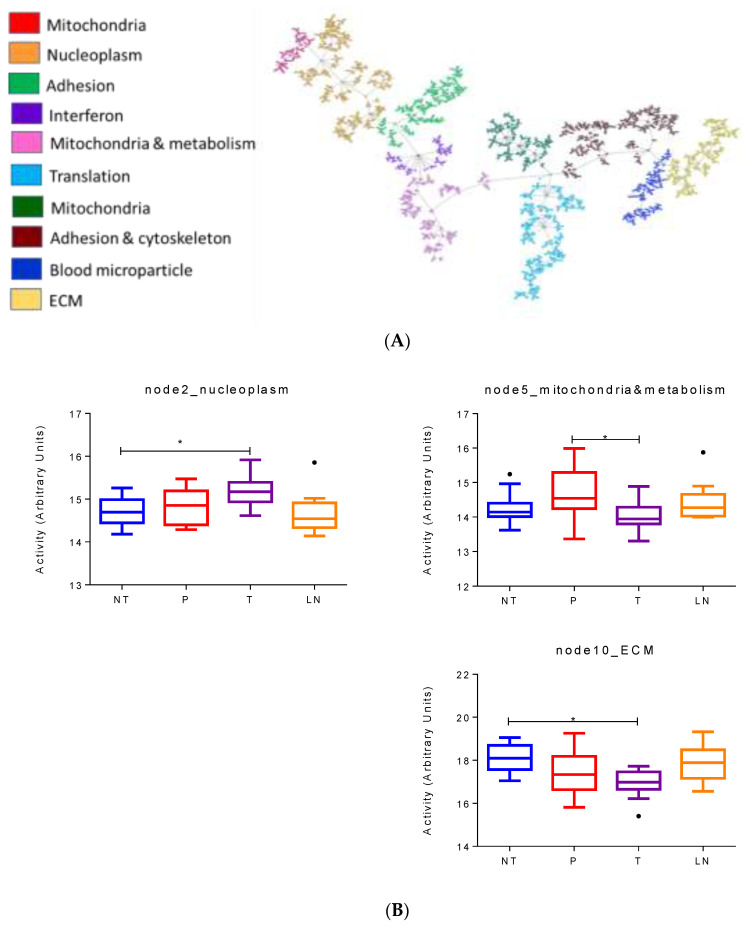
(**A**) Network of 2311 proteins in the T3 subtype. (**B**) Differential functional node activities comparing the different histological samples in the T3 subtype according to mixed lineal models. NT = normal tissue, P = preneoplastic lesions, T = primary tumors, LN = lymph nodes. * *p* < 0.05.

T3 subtype was characterized by higher nucleoplasm activity and also had overlapping characteristics with the classical subtype. The obtained network using protein expression data from the T3 samples was composed of ten functional nodes (Figure 5A).

Functional node activities showed differences between no tumor and tumor samples in nucleoplasm, mitochondria and metabolism, and ECM. ECM had a decrease in their activity in tumors compared to normal samples, while nucleoplasm showed an increase in tumor samples. In addition, tumor samples presented a decrease in mitochondria and metabolism node activity compared to PanIN. In the case of tumor and lymph nodes, there are no significantly different processes between them (Figure 5B).

Nucleoplasm nodes were formed by some well-known proteins, such as PARP1, ELAVL1, SART3, RAN, FUBP1, APEX1, or AKT1S1.

A summary of the differential functional node activities and their corresponding biological processes is presented in Table 2. Complete results of the mixed linear models are provided in Supplementary Figure S1.

### 3.6. Validation of These Tumor Subtypes in the TCGA Cohort

To confirm the described PDAC proteomics subtypes, the TCGA cohort was used. According to a centroid assignation, there were 46 (25%) PDAC samples in the T1 subtype, 73 (40%) in the T2 subtype, and 65 (35%) samples in the T3 subtype. Using functional node activities calculated in this cohort confirmed that the TCGA samples assigned to the T2 subtype had metabolic characteristics. The T1 subtype samples had higher activities in adhesion nodes as it occurred in the proteomics cohort. T3 subtype showed a higher activity in nucleoplasm and splicing functional nodes (Supplementary Figure S4). No differences in overall survival between the tumor proteomics subtypes were found.

## 4. Discussion

This is the first study in PDAC using proteomics to define the molecular subtypes and mechanisms involved in tumor development and progression in each subtype. Samples from 52 PDAC patients, including non-tumor tissues, preneoplastic lesions, primary tumors, and lymph nodes, were analyzed by high-throughput proteomics and a Systems Biology approach in order to identify relevant biological processes in tumor development and progression.

Using this approach, we have defined three proteomics PDAC subtypes, which can be detected even in the earliest stages of tumor development. Each subtype showed specific molecular features. T1 subtype is related to adhesion, the T2 subtype has metabolic features, and the T3 subtype presents high splicing and nucleoplasm activity. These proteomics subtypes also shared some characteristics with subtypes previously defined by transcriptomics, while providing new and complementary information: T2 tumors correspond to ADEX subtype, including some metabolic basal-like and classical subtypes; T1 contained basal-like and classical subtypes; and T3 corresponded to those classical tumors with high expression of nucleoplasm-related proteins. Interestingly, the identified processes involved in tumor development and progression were different between the three PDAC proteomics subtypes, suggesting that the molecular motor of the disease is different in each subtype. These differences can have implications for the development of future tailored therapeutic approaches for each PDAC proteomics subtype.

Previous transcriptomics studies defined a group of tumors in which adhesion plays an important role [4,5,6], as observed in our proteomics subtype T1. Carcinogenesis in the T1 subtype seems to be related to a decrease of mitochondria, pancreatic secretion and translation node activity, and an increase of adhesion and complement activation and antigen presentation node activity. The adhesion 2 functional node contains some relevant proteins, such as HSPB1 and THY1. The HSPB1 gene codifies Heat Shock Protein 27 (Hsp27), a cell survival protein found at elevated levels in many human cancers, including prostate, lung, breast, ovarian, bladder, renal, pancreatic, multiple myeloma, and liver [22,23]. THY1, also known as CD90, is a stem cell marker that interacts with monocytes and macrophages, promoting immunosuppressive features of immune cells and enhancing the stemness and E-MT of PDAC. It has been suggested that THY1 establishes a favorable environment that promotes tumor progression [24], which can be mediated by high levels of PD-L1 in CD90^+^ cells [25]. In addition, complement and antigen activation functional nodes are mainly composed of immunoglobulins and complement proteins. Although the role of complement in PDAC development is still unclear [26], the role of complement in tumor development and modulation of the tumor microenvironment has been demonstrated [27]. The expression of complement C3 in pancreatic cancer was described as significantly higher than in normal tissues and was proposed as a diagnostic biomarker of early-stage pancreatic cancer [28,29]. Depletion of C3 in tumor cells enhanced the efficacy of anti–PD-L1 treatment [30]. These results together suggest that high levels of THY1 and complement components in T1 tumors provoke an immunosuppressive tumor microenvironment, suggesting an inflammatory phenotype, and open up the possibility of using a combination of immunotherapy coupled with anti-PD1/PD-L1 therapy in patients with these T1 tumors. Differences between tumors and lymph nodes in the T1 subtype, related to tumor progression, were identified in nucleoplasm, translation, adhesion, extracellular matrix, and complement activation nodes. In the nucleoplasm functional node, HIF1AN stands out due to its role in the regulation loop of IGFR. It has been described that the use of an IGFR inhibitor caused a lower expression of this protein and a decrease in growth in pancreatic cancer cells [31,32] Thus, IGFR pathway inhibitors may avoid tumor progression in PDAC T1 proteomics subtype.

Our data in primary tumors confirmed that mitochondria metabolism plays an important role in one of the PDAC proteomics subtypes, the T2 subtype. Our analysis based on probabilistic graphical models also highlighted the importance of glycolysis and pyruvate metabolism, valine metabolism, and fatty acid metabolism, among others. In a previous study analyzing tumors and adjacent tissue from three PDAC patients, differential proteins related to metabolism, especially mitochondrial proteins and proteins whose function is acting as regulators of pancreatic juices, were identified [33]. The importance of metabolic alterations in PDAC, including an increase in glutamine metabolism and mitochondrial dysfunction, has been previously highlighted [34]. In addition, in a previous proteomics study in hepatic PDAC metastases, a group related to metabolism was defined, characterized by the expression of ethanol oxidation, mitochondrial fatty-acid beta oxidation, and retinoic acid signaling pathways [9]. Moreover, the downregulation of certain metabolic pathways in patients with PDAC and diabetes mellitus has been suggested to be associated with the poor prognosis of these patients [35]. Metabolism and pancreatic secretion nodes had differential activity between T2 normal tissue, PanIN, and tumors and between tumors and lymph nodes. Pancreatic secretion node contained some relevant proteins. For instance, expression of the angiogenic factor TYMP has been correlated with capecitabine and fluorouracil response [36,37]. Additionally, in their proteomics study, Law et al. established that TYMP had a strong correlation with patient survival in PDAC [9]. Another protein in this node is NAMPT, whose inhibitor STF-118804, in combination with chemotherapy agents, such as paclitaxel, gemcitabine, and etoposide, showed an additive effect in the decrease of cell viability and growth in PDAC [38]. PNLIP is one of the main pancreatic lipases. It is related to orlistat, a drug used in obesity treatment. Kridel et al. stated that orlistat may inhibit the growth of prostate cancer by interfering with the metabolism of fats [39]. Interestingly, in lymph node metastases, these processes presented a significant decrease compared to primary tumors. In conclusion, regarding the T2 subtype, it seems that metabolism and, especially, mitochondria act as the motor of cancer development.

The last proteomics subtype, T3, is related to nucleoplasm and histones. Mutational studies of PDAC showed a high prevalence of genetic alterations in genes involved in chromatin remodeling such as *SMARCA2, SMARCA4, MLL2,* or *ARID1A*, among others [40], so it is not surprising that proteomics subtyping highlighted the relevance of proteins related to nucleoplasm and histone modification. This group was also GATA6 positive, being equivalent to classical PDAC tumors. T3 analyses point out that nucleoplasm, mitochondria and metabolism, and extracellular matrix nodes could be involved in T3 tumor carcinogenesis. The nucleoplasm node also contained some well-known cancer-related proteins, such as PARP1. ELAVL1 is also present in this functional node, and it has been associated with response to gemcitabine in pancreatic cancer [41]. SART3 is an RNA-binding nuclear protein that is a tumor-rejection antigen. This antigen possesses tumor epitopes capable of inducing HLA-A24-restricted and tumor-specific cytotoxic T lymphocytes in cancer patients and may be useful for specific immunotherapy. RAN promotes metastasis and invasion in pancreatic cancer by deregulating the expression of AR and CXCR4. In this study, they also demonstrated that the expression of Ran was remarkably higher in lymph lode metastases than in primary pancreatic cancer tissue [42]. FUBP1 is a target of irofulven, a novel anti-cancer compound whose anti-tumor activity in an advanced pancreatic cancer patient was documented [43]. The APEX1 redox selective inhibitor E3330 caused a significant inhibition of tumor cell migration in PDAC [44]. AKT1S1 is a target of rapamycin, a drug used in the treatment of other cancers [45,46].

Proteomics has been previously used to characterize PDAC disease, employing serum, pancreatic juice, fresh tissue, and paraffin samples. Holm et al. analyzed 21 serum samples from patients with pancreatic cancer to identify proteins differentially expressed in patients with long or short survival [47]. Paulo et al. compared PanIN lesions and PDAC FFPE samples, identifying a list of exclusive proteins for each condition. Annexin 4A, fibronectin and mucin 2 were exclusively expressed in PDAC samples [48]. Naidoo et al. conducted the first study of FFPE samples comparing PDAC and lymph node metastases and found that differentially expressed proteins were mostly related to the immune system and metabolic processes [49]. Cao et al. recently identified some proteins that could be useful as early detection biomarkers in PDAC comparing normal and tumor tissue [11]. However, in these studies, the differences between molecular subtypes were not evaluated. In this context, our approach has two main advantages: first, analyzing different stages of tumor progression (non-tumor tissue, PanIN, tumor, and lymph nodes) allowed us to study carcinogenesis (differences between PanIN and tumor tissue) and tumor dissemination (differences between tumor tissue and lymph nodes) independently. Second, our analytical pipeline allows studying biological processes instead of proteins individually, providing a naive and undirected context to the high-throughput proteomics data and allowing interpretation of the molecular features detected in each proteomics subtype. Additionally, our proteomic subtypes were validated by the PDAC TCGA cohort.

Remarkably, our analyses showed that differences between tumor subtypes are higher than between types of tissues. Connor et al. analyzed 19 paired samples, primary tumors, and metastases, and showed that they were molecularly conserved, i.e., paired metastases and primary tumors were classified in the same molecular subtype [50]. The fact that adjacent non-tumor tissue is more related to its neighbor tumor than non-tumor tissue from other patients suggests that the physical tumor border does not correspond with the molecular tumor border in PDAC.

Drug development in PDAC is challenging, as modest results of immunotherapy in this pathology point out. Although several reasons for this lack of results have been proposed [51], the inclusion of unselected patients in clinical trials, regarding its molecular features, may be a hidden factor, pointing out the need to consider molecular heterogeneity of PDAC in future developments. Our results suggest some therapeutic strategies to follow up on in each proteomics subtype. For instance, regarding T1 tumors, HSPB1 is the target of the drug apatorsen, a second-generation antisense drug able to inhibit the production of Hsp27 in preclinical experiments. Data from the RAINIER trial showed that adding apatorsen to gemcitabine+nab-paclitaxel did not improve the outcome of unselected metastatic PDAC patients, but can be useful in those patients with high serum doses of Hsp27 [52]. Additionally, high levels of THY1/PD-L1 and complement components in T1 tumors provoke an immunosuppressive tumor microenvironment, suggesting an inflammatory phenotype [25], and depletion of C3 in tumor cells enhanced the efficacy of anti–PD-L1 treatment [30]. These results open up the possibility of using a combination of complement immunotherapy coupled with anti-PD1/PD-L1 therapy in patients with T1 tumors. Regarding T2 tumors, mitochondria are emerging as an interesting actionable target, with numerous clinical trials currently testing different drugs modulating mitochondrial activity in PDAC [53]. Finally, T3 tumors showed overexpression of a variety of actionable targets. Veliparib, a PARP-1/2 inhibitor, was tested with gemcitabine and radiotherapy in locally advanced pancreatic cancer in a phase 1 study, and the results supported a phase 2 validation study [54].

The main limitation of this study was the impossibility of obtaining all types of samples from each patient. This limitation was mitigated using linear mixed models. Additionally, after dividing samples by subtype, the number of samples in each group decreased, which may have prevented the detection of differences in the possible predisposing factors, clinical characteristics, and prognosis of the different proteomics subtypes. In addition, all biological processes that might be therapeutic targets in the future need further study.

## 5. Conclusions

In this study, three PDAC proteomics subtypes were defined: an adhesion-related subtype (T1), a metabolic-related subtype (T2), and a nucleoplasm subtype (T3). We also suggested several biological processes involved in tumor development and progression characteristics of each proteomics subtype, suggesting that the motor of the disease is different in each subtype. These biological processes could be relevant as a guide to stratify patients and select candidates for future tailored therapeutic treatments in PDAC.

## Figures and Tables

**Table 1:** Patients’ characteristics.

	Number of Patients = 50 (100%)
**Gender**	
Male	30 (60%)
Female	20 (40%)
**Age (median) (IQR)**	28–84 (65) (52–76)
**Diabetes at diagnosis**	
Yes	9 (18%)
No	40 (80%)
Unknown	1 (2%)
**Tobacco use**	
Yes	22 (44%)
No	21 (42%)
Unknown	7 (14%)
**Location of primary tumor**	
Head	38 (76%)
Body	3 (6%)
Tail	5 (10%)
Various	4 (8%)
**Grade**	
Very differentiated	6 (12%)
Moderately	33 (66%)
Poor	8 (16%)
Unknown	3 (6%)
**Type of resection**	
R0	16 (32%)
R1	34 (68%)
**pT**	
1	3 (6%)
2	11 (22%)
3	34 (68%)
4	2 (4%)
**pN**	
N0	11 (22%)
N1	39 (78%)
**Stage (TNM 7th edition)**	
Ia	2 (4%)
Ib	2 (4%)
IIa	4 (8%)
IIb	38 (76%)
III	3 (6%)
IV	1 (2%)

**Table 2:** Summary of functional node activities identified as differential using mixed linear models between samples in each PDAC proteomics subtype. NT = no tumor tissue, P = preneoplastic lesions, T = primary tumor, LN = lymph node metastasis.

Samples	Direction	T1	T2	T3
NT→T	↓			ECM
	↑		Pancreatic secretion Metabolism	Nucleoplasm
P→T	↓	Pancreatic secretion Mitochondria Translation		Mitochondria & metabolism
	↑	Complement activation Adhesion 2	Pancreatic secretion Metabolism	
T→LN	↓	Complement activation Adhesion 2 ECM	Pancreatic secretion Metabolism	
	↑	Nucleoplasm Translation		

## Data Availability

All relevant data have been deposited to the ProteomeXchange Consortium via the PRIDE (http://www.ebi.ac.uk/pride) partner repository with the data set identifier PXD032076.

## Supplementary Materials

The following supporting information can be downloaded at: https://www.mdpi.com/article/10.3390/cancers14102414/s1, Figure S1: A. Distribution according to the location of primary tumor in PDAC proteomics subtypes. B. Disease-free survival (DFS) and overall survival (OS) according to the three PDAC proteomics subtypes; Figure S2: Mucin-5 (MUC5A) and insulin (INS) expression in PDAC proteomics subtypes. ***: *p* < 0.0001; **: 0.0001 < *p* < 0.001; *: 0.001 < *p* < 0.05; *p* < 0.05; Figure S3: Distribution of GATA6 immunohistochemical expression in PDAC proteomics subtypes; Figure S4: Validation of the functional node activities in the TCGA cohort. ***: *p* < 0.0001; **: 0.0001 < *p* < 0.001; *: 0.001 < *p* < 0.05; *p* < 0.05.
