# Supplementary material for: Identification of Carcinogenesis and Tumor Progression Processes in Pancreatic Ductal Adenocarcinoma Using High-Throughput Proteomics
Source: Cancers (Basel). 2022 May 13;14(10):2414. doi: 10.3390/cancers14102414 (PMC9139847; doi:10.3390/cancers14102414)
Supplement: Supplementary file 1 [file cancers-14-02414-s001.zip › cancers-1713131-supplementary.pdf]

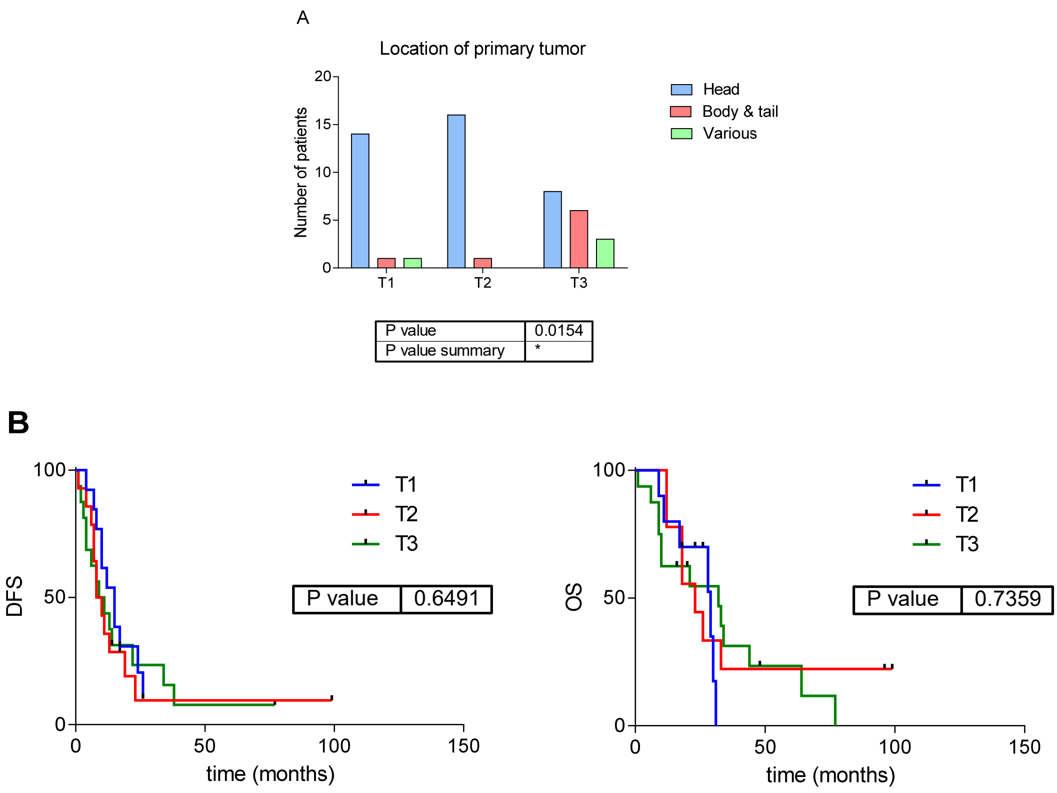

Supplementary Figure S1: A. Distribution according to the location of primary tumor in PDAC proteomics subtypes. B. Disease-free survival (DFS) and overall survival (OS) according to the three PDAC proteomics subtypes.

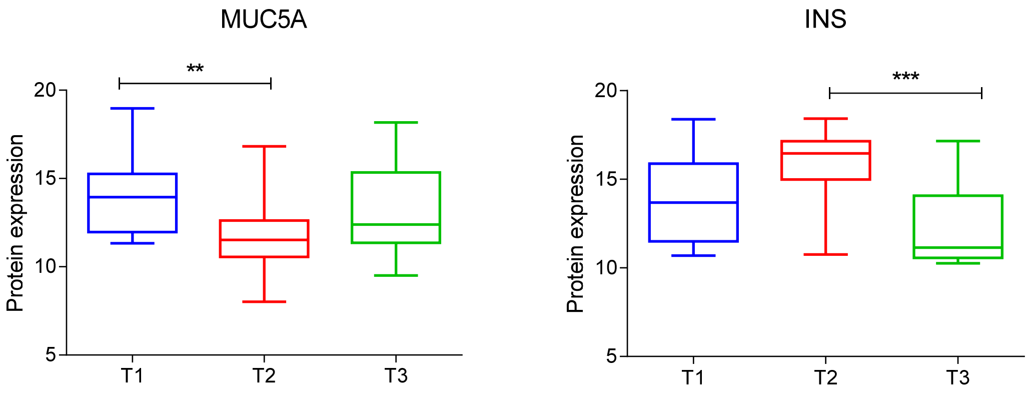

Supplementary Figure S2: Mucin-5 (MUC5A) and insulin (INS) expression in PDAC proteomics subtypes. ****: p<0.0001; ***: 0.0001<p<0.001; **: 0.001<p<0.05; *: p<0.05.

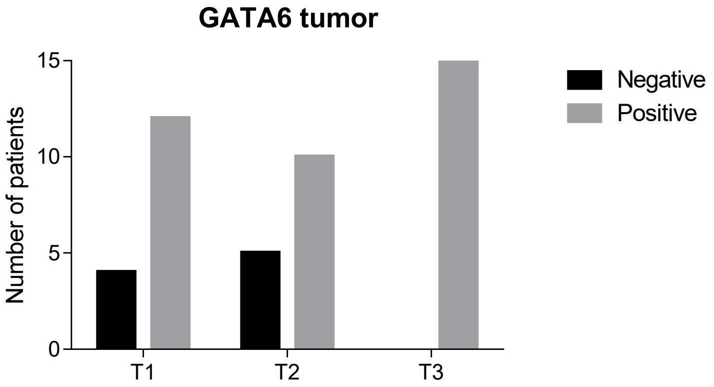

Supplementary Figure S3: Distribution of GATA6 immunohistochemical expression in PDAC proteomics subtypes.

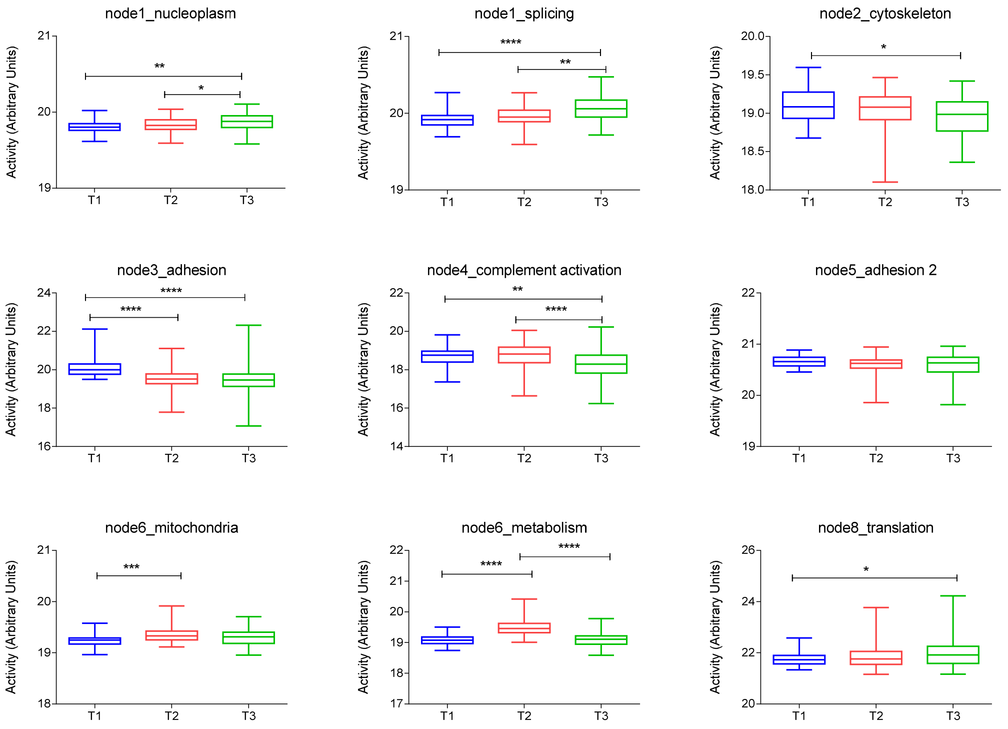

Supplementary Figure S4: Validation of the functional node activities in the TCGA cohort. ****: p<0.0001; ***: 0.0001<p<0.001; **: 0.001<p<0.05; *: p<0.05
